# Cilostazol combined with P2Y_12_ receptor inhibitors: A substitute antiplatelet regimen for aspirin‐intolerant patients undergoing percutaneous coronary stent implantation

**DOI:** 10.1002/clc.23787

**Published:** 2022-02-04

**Authors:** Yikai Zhao, Peng Zhou, Wen Gao, Haoxuan Zhong, Yufei Chen, Wei Chen, Maieryemu Waresi, Kun Xie, Haiming Shi, Hui Gong, Guibin He, Zhaohui Qiu, Xinping Luo, Jian Li

**Affiliations:** ^1^ Department of Cardiology Huashan Hospital of Fudan University Shanghai People's Republic of China; ^2^ Department of Cardiology Jinshan Hospital of Fudan University Shanghai People's Republic of China; ^3^ Department of Cardiology Luodian Hospital Shanghai People's Republic of China; ^4^ Department of Cardiology Tongren Hospital of Shanghai Jiao Tong University Shanghai People's Republic of China

**Keywords:** antiplatelet therapy, aspirin intolerance, cilostazol, percutaneous coronary stent implantation, platelet activation

## Abstract

**Background:**

Cilostazol combined with P2Y_12_ receptor inhibitor has been used as a substitute regimen for aspirin‐intolerant patients undergoing percutaneous coronary stent implantation on a small scale. Its exact impact on platelet functions and clinical benefits of aspirin‐intolerant patients is unknown.

**Hypothesis:**

Cilostazol combined with P2Y_12_ receptor inhibitors could be used as a substitute antiplatelet regimen for aspirin‐intolerant patients undergoing percutaneous coronary stent implantation.

**Methods:**

In this multicenter prospective cohort trial, patients undergoing elective percutaneous coronary stent implantation were assigned to the cilostazol group (cilostazol plus P2Y_12_ receptor inhibitors), based on aspirin intolerance criteria, or the aspirin group (aspirin plus P2Y_12_ receptor inhibitors). Platelet PAC‐1, CD62p, and vasodilator‐stimulated phosphoprotein phosphorylation (VASP‐P) were detected by flow cytometry. The primary endpoints were major adverse cardiovascular and cerebrovascular events (MACCE) including all‐cause death, acute myocardial infarction, emerging arrhythmia, nonfatal stroke, and heart failure. The secondary endpoints were the Bleeding Academic Research Consortium (BARC) bleeding events.

**Results:**

One hundred and fifty‐four aspirin‐intolerant percutaneous coronary stent implantation patients and 154 matched aspirin‐tolerant patients from a total of 2059 percutaneous coronary stent implantation patients were enrolled. The relative activation level of PAC‐1, CD62p, and platelet reaction index reflected by the VASP‐P test were similar in the two groups (*p* > .05). After 12 months of follow‐up, the incidence of all‐cause death was 1.9% in the cilostazol group and 1.3% in the aspirin group (risk ratio [RR], 1.500; 95% confidence interval [CI], 0.254–8.852; *p* = 1.000); the incidence of acute myocardial infarction was 0.6% in the cilostazol group and 1.3% in the aspirin group (RR, 0.500; 95% CI, 0.046–5.457; *p* = 1.000). No significant difference was seen in other MACCE events, or in any types of BARC bleeding events.

**Conclusions:**

Cilostazol combined with P2Y_12_ inhibitors was not inferior to aspirin‐based standard therapy and could be used as a reasonable substitute antiplatelet regimen for aspirin‐intolerant patients undergoing percutaneous coronary stent implantation, but again with limitations, which required a larger sample and longer follow‐up to confirm its efficacy.

## 1 INTRODUCTION

Dual antiplatelet therapy consisting of aspirin and P2Y_12_ receptor inhibitors has become a standard treatment to prevent thrombotic complications for patients undergoing percutaneous coronary stent implantation.^1^, ^2^ However, aspirin‐intolerant patients undergoing stent implantation with previous peptic ulcer, erosive gastritis, gastrointestinal bleeding, gout, and aspirin‐related mucocutaneous or respiratory hypersensitivity were facing a higher risk of aspirin‐related adverse reactions, which might outweigh the antiplatelet benefit of standard dual antiplatelet therapy (DAPT).^3^, ^4^, ^5^ Several measures have been attempted to deal with the dilemma, but neither adding proton pump inhibitors nor aspirin desensitization could address aspirin intolerance situation.^6^, ^7^, ^8^

Cilostazol acts as an antiplatelet drug by selectively inhibiting the activity of phosphodiesterase III, further inhibiting the decomposition of cAMP.^9^ Since cilostazol does not affect the cyclooxygenase pathway, it exerts an antiplatelet effect without increasing the risk of gastrointestinal bleeding and other adverse reactions relating to aspirin.^10^ Nonaspirin‐based DAPT, such as cilostazol combined with P2Y_12_ receptor inhibitors, has been used for aspirin‐intolerant patients on a small scale.^11^ However, the efficacy and safety, especially the antiplatelet effects, have not been truly verified by large‐scale clinical trials.

To this end, we designed a multicenter prospective nonrandomized controlled trial to investigate whether cilostazol combined with P2Y_12_ receptor inhibitor was not inferior to aspirin based DAPT.

## 2 METHODS

### 2.1 Patients

This multicenter prospective cohort trial enrolled successive patients undergoing elective percutaneous coronary stent implantation in four clinical centers (Huashan Hospital, Jinshan Hospital, Tongren Hospital, and Luodian Hospital) in Shanghai, China from January 2018 to January 2020. The trial was conducted in compliance with the Declaration of Helsinki and Good Clinical Practice. The study protocol was approved by ethics committees or institutional review boards at participating sites; patients provided written informed consent before inclusion in the study.

The inclusion criteria included (1) age between 18 and 85 years old; (2) diagnosed with coronary heart disease and underwent elective percutaneous coronary stent implantation from January 2018 to January 2020; (3) PRECISE‐DAPT score <25 (low bleeding risk).^12^ The exclusion criteria included (1) age younger than 18 years or older than 85 years; (2) PRECISE‐DAPT score ≥25; (3) contraindications of clopidogrel, ticagrelor, or cilostazol; (4) stent implanted within 1 year before admission, coronary artery bypass graft was performed within 2 months; (5) stroke or cerebral hemorrhage; (6) antiplatelet and antifibrin drugs other than aspirin, clopidogrel, ticagrelor, or cilostazol were taken within 5 days before percutaneous coronary stent implantation; (7) patients with atrial fibrillation or patients undergoing oral anticoagulation therapy; (8) hemoglobin (Hb) < 10 g/L; (9) transaminase two times higher than the upper limitation; (10) oncological patients; (11) severe bleeding tendency, severe anemia, thrombocytopenia; (12) pregnancy; (13) mental illness; (9) inability to follow the protocol.

### 2.2 Grouping and treatment

Patients were divided into the cilostazol group and the aspirin group according to the assessment of whether patients could tolerate aspirin at admission. Since there has never been a formal definition of “aspirin intolerance” before, we redefined the clinical criterion based on previous cross‐sectional investigations and related cohort trials.^11^, ^13^ “Aspirin intolerance” was defined as (1) aspirin‐related gastrointestinal discomforts like acid reflux, vomiting, abdominal pain; (2) history of peptic ulcer, atrophic gastritis, erosive gastritis, antral gastritis, and reflux esophagitis; (3) gastrointestinal bleeding history or comorbid high bleeding risk, positive fecal occult blood during hospitalization (at least consecutive two times); (4) history of subtotal gastrectomy or gastric polypectomy within 3 years; (5) hyperuricemia/gout; (6) aspirin‐exacerbated respiratory disease including severe rhinitis, bronchospasm, and aspirin‐induced respiratory diseases^14^; (7) aspirin‐related skin and mucosal hypersensitivity reactions including rash, urticaria, edema, and allergic shock. Patients who met any aspirin intolerance criteria above were assigned to the cilostazol group (cilostazol plus clopidogrel or ticagrelor; the maintaining dose was 50 mg bid, 75 mg qd, 90 mg bid for cilostazol, clopidogrel, and ticagrelor, respectively). Once a patient was enrolled into the cilostazol group, one aspirin‐tolerant patient would be matched into the aspirin group by propensity score matching in a 1:1 ratio to ensure comparability between groups. The maintaining dose was 100 mg qd, 75 mg qd, 90 mg bid for aspirin, clopidogrel, and ticagrelor, respectively, in the aspirin group.

### 2.3 Platelet collection and flow cytometry

Patients' venous blood after 3 days of antiplatelet administration was collected to compare the antiplatelet effects of different regimens by flow cytometry. Laboratory measurements were performed within 2 h of peripheral blood sampling. Platelets were isolated and incubated with or without 1 μl ADP (2 mmol/L), then stained with 1 μl of each antibody (phycoerythrin‐conjugated anti‐human CD61 monoclonal antibody, fluorescein isothiocyanate‐conjugated anti‐human PAC‐1 monoclonal antibody, and APC AK‐4 CD62p monoclonal antibody). CD62P reflected the expression of platelet P‐selectin, while PAC‐1 reflected the level of activated platelet glycoprotein GPIIb/IIIa fibrinogen receptor.^15^, ^16^, ^17^, ^18^ Meanwhile, platelet vasodilator‐stimulated phosphoprotein phosphorylation (VASP‐P) was measured to detect the platelet VASP phosphorylation level, which reflected the platelet reaction index (PRI) indirectly.^19^, ^20^ VASP‐P measurement followed the protocol provided by the Biocytex kit.

### 2.4 Follow‐up and endpoints

The primary endpoint (efficacy endpoint) was the first occurrence of major adverse cardiovascular events (MACCE), including all‐cause death, acute myocardial infarction, emerging arrhythmia, nonfatal stroke, heart failure. The secondary endpoint (safety endpoint) was Bleeding Academic Research Consortium (BARC) bleeding events following BARC standards and other skin/mucosal adverse reactions (rash, urticaria, conjunctivitis, angioedema), headache, and gastrointestinal discomfort.^21^ All outcomes were adjudicated according to standard definitions by an independent committee blinded to treatment assignment. All enrolled patients were followed 12 months after discharge. Endpoints were acquired through telephone interviews and outpatient clinics。

### 2.5 Statistical analysis

Baseline characteristics were summarized for the two groups by treatment allocation. The rate of MACCE was estimated to be 13% in the cilostazol group in 1 year of follow‐up. The study would have 80% power to detect the difference with a two‐sided *α* level of .05. The sample size was estimated to be 132 in each group. Assuming a dropout rate of 10%, a total of 145 patients in each group were required. Propensity score matching was used to account for the differences in baseline characteristics between the cilostazol group and the aspirin group.^22^ Categorical variables were compared by paired McNeill–Marr *χ* ^2^ test or Fisher exact test. Continuous variables were compared by paired Student *t* test or paired Wilcoxon rank test (nonnormally distributed data). Significant MACCE events and severe bleeding events were analyzed by survival analysis, using the Kaplan–Meier method and the log‐rank test to analyze the survival curves between groups; if multiple factors might affect survival time (between groups), Cox proportional hazards regression model would be used for further processing.^23^ *P* value less than .05 was considered to be statistically different. Statistical analyses were performed using SPSS 26.0 software and PRISM 8.

## 3 RESULTS

### 3.1 Baseline characteristics

**Figure 1:**
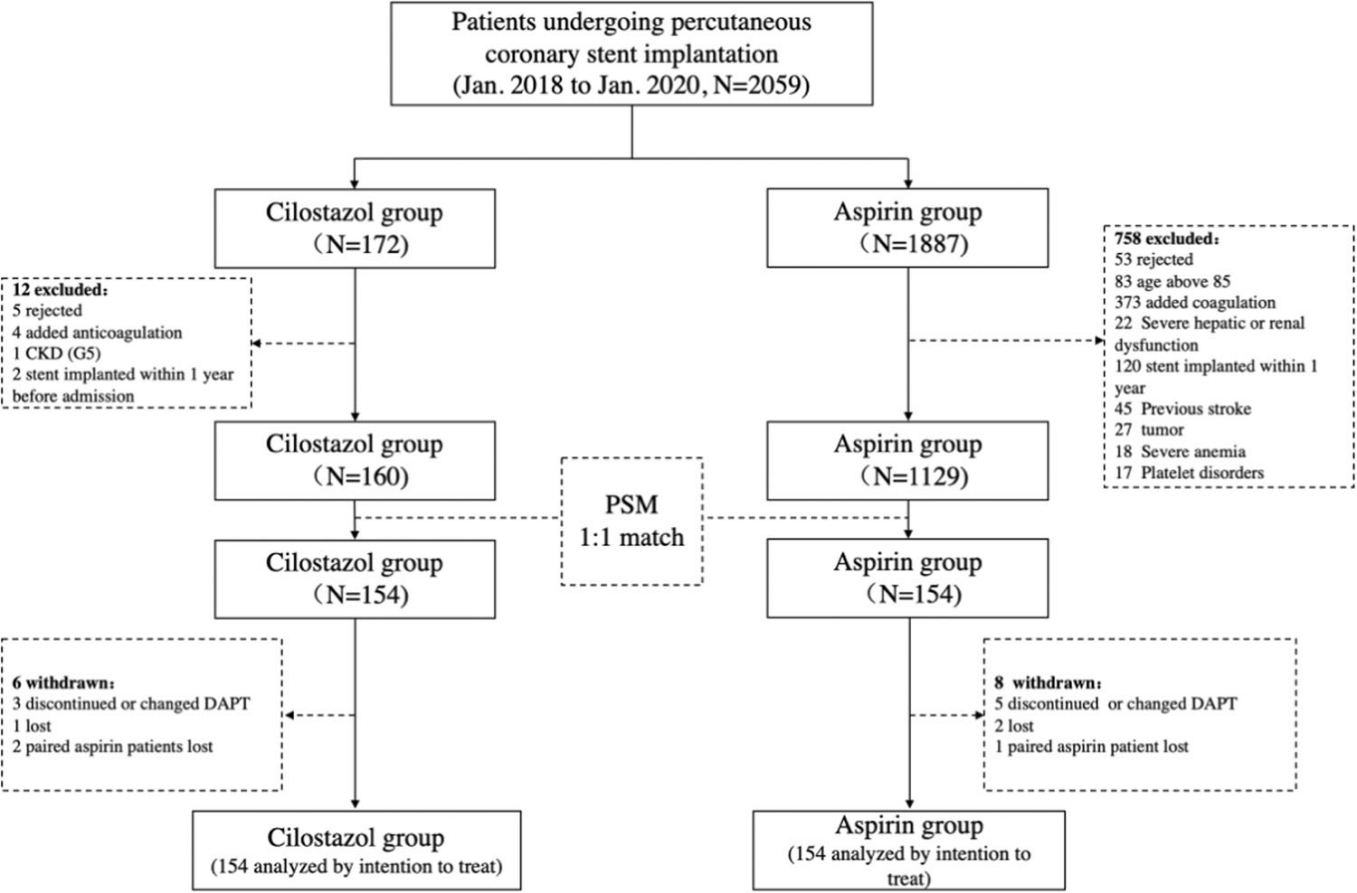
Flowchart of the enrollment, matching and follow‐up of the study population. CKD, chronic kidney disease; DAPT, dual antiplatelet therapy; PSM, propensity score matching

From January 2018 to January 2020, a total of 2059 patients underwent elective percutaneous coronary stent implantation. Among them, 172 patients were given cilostazol combined with P2Y_12_ receptor inhibitors due to a clear reason for aspirin intolerance. Of the 172 patients, 160 met our inclusion criteria and entered the cohort. In the initial multivariable analysis, variables consisting of age, chronic kidney disease, smoking history, low‐density lipoprotein cholesterol, and P2Y_12_ receptor inhibitor were found with significant differences between the two groups. After 1:1 propensity score matching, a total of 154 patients in the cilostazol group and matched 154 patients in the aspirin group successfully entered the final comparison of follow‐up (Figure 1 and Table S1), and all baseline characteristics were well‐balanced between the two groups (Table 1). Meanwhile, 96 patients' venous blood (1:1 paired from the two groups) were collected for antiplatelet effect detection by flow cytometry after informed consent.

**Table 1:** Baseline characteristics of enrolled patients

Characteristic	Cilostazol (*N* = 154)	Aspirin (*N* = 154)	*p* value
Alcohol	28	27	1.000
Previous PCI	28	25	.749
PLT	198.5 (149.0–237.3)	197 (151.75–239)	.694
MPV	10.8 (10–11.8)	10.7 (10.08–11.23)	**.046**
CK‐MB	2.22 (1.47–4.62)	2.085 (1.51–3.66)	.578
cTNT	0.065 (0.01–0.28)	0.02 (0.01–0.15)	.155
Pro‐BNP	303.8 (81.38–790.3)	191 (55.73–659.93)	.443
hsCRP	2.6 (0.63–7.00)	1.52 (0.61–4.10)	**.029**
eGFR	82.55 (60.0–97.16)	86.78 (69.94–108.67)	**.001**
HbA1c	6.1 (5.7–6.9)	6 (5.6–7.0)	.680
TC	3.84 (3.07–4.46)	4.02 (3.36–4.65)	.058
TG	1.27 (0.89–1.79)	1.41 (1.07–2.04)	**.027**
Coagulation function			
APTT	26.95 (23.43–29.85)	26.3 (23.58–29.73)	.886
TT	17.85 (17.10–18.73)	18.1 (17.5–19.0)	.099
PT	11.45 (11.0–12.13)	11.3 (10.8–11.8)	**.032**
Fib	3.1 (2.5–3.9)	2.9 (2.5–3.4)	.098
D‐dimer	0.375 (0.20–0.67)	0.32 (0.19–0.49)	**.003**
Administration			
Statin	138 (90)	144 (94)	.327
CCB	39 (25.3)	62 (40.3)	**.007**
β‐blockers	104 (67.5)	100 (64.9)	.712
ACEI/ARB	101 (65.6)	97 (63.0)	.720
Nitrate	58 (37.7)	52 (33.8)	.550
Results of angiography			
No. of stents	1.4 ± 0.3	1.8 ± 0.2	.283
Lesion			
LM	5	12	.062
LAD	65	72	.123
D	8	10	.259
LCX	49	59	.082
OM	15	11	.072
RCA	37	52	.051
PLA	5	3	.928
PDA	1	1	1.000

*Note*: Variabled with bold P values were statistically different between the two groups in baseline. To clarify whether these covariables had an impact on endpoints rates, Cox proportional hazards regression was further conducted (see 3.5 Survival analysis).

Abbreviations: ACEI, angiotensin‐converting enzyme inhibitor; APTT, activated partial thromboplastin time; ARB, angiotensin receptor blocker; CCB, calcium channel blocker; CK‐MB, creatine kinase MB; cTNT, cardiac troponin T; eGFR, estimated glomerular filtration rate; Fib, fibrinogen; HbA1c, hemoglobin A1c; hs‐CRP, high‐sensitivity C‐reactive protein; LAD, left anterior descending; D, diagonal branches; LCX, left circumflex artery; LM, left main; MPV, mean platelet volume; OM, obtuse marginal branch; PCI, percutaneous coronary intervention; PDA, posterior descending artery; PLA, posterolateral artery; PLT, platelet; pro‐BNP, probrain natriuretic peptide; PT, prothrombin time; RCA, right coronary artery; TC, total cholesterol; TG, triglycerides; TT, thrombin time; UA, urine acid; LM.

### 3.2 Flow cytometry test

**Figure 2:**
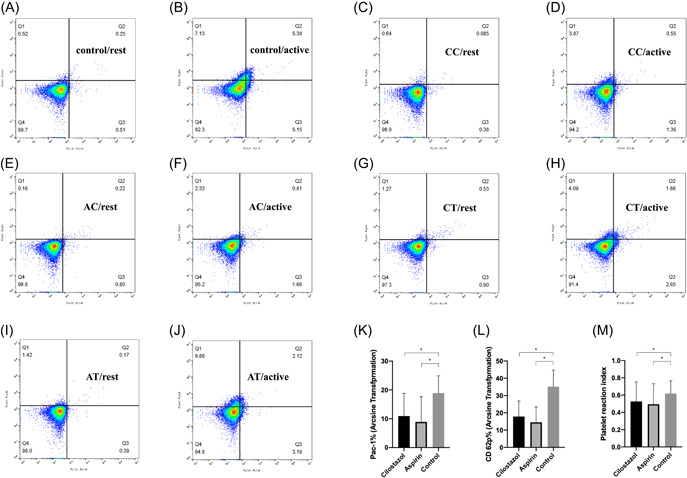
(A–J) The relative activation level of PAC‐1 and CD62p in the control (placebo) and cilostazol and aspirin groups. (K and L) The differences of PAC‐1 and CD62p between groups in histograms, (M) the difference of PRI tested by vasodilator‐stimulated phosphoprotein phosphorylation (VASP‐P) flow cytometry. Horizontal axis, PAC‐1; vertical axis, CD62p; AC, aspirin plus cilostazol; AT, aspirin plus cilostazol; CC, cilostazol plus clopidogrel; CT, cilostazol plus ticagrelor; rest, incubated without ADP; active, incubated with ADP

Forty‐eight patients in the cilostazol group and 48 matched patients in the aspirin group participated in this study part. The relative activation level of PAC‐1 in the cilostazol group was 10.95 ± 7.85%, the relative activation level of PAC‐1 in the aspirin group was 9.09 ± 8.09%. There was no significant difference between the two groups (*p* = .171). The relative activation level of platelet CD62p in the cilostazol group was 17.87 ± 8.97%, which was 14.51 ± 8.87% in the aspirin group (*p* = .055) (Figure 2). The PRI measured by the VASP‐P method in the cilostazol group was 52.60 ± 22.58%, which was similar to that of the aspirin group (49.51 ± 23.76%, *p* = .507) (Table S2).

### 3.3 MACCE

The primary endpoint of MACCE during 12 months occurred in 22 patients (14.3%) in the cilostazol group and 23 patients (14.9%) in the aspirin group. The incidence of acute myocardial infarction events was 0.6% in the cilostazol group and 1.3% in the aspirin group (risk ratio, 0.500; 95% confidence interval [CI], 0.046–5.457; *p* = 1.000); All‐cause death occurred 1.9% in the cilostazol group and 1.3% in the aspirin group (risk ratio, 1.500; 95% CI, 0.254–8.852; *p* = 1.000). There were no significant differences between the two groups in emerging arrhythmia (risk ratio, 0.667; 95% CI, 0.192–2.316; *p* = .727), nonfatal stroke (risk ratio, 2.000; 95% CI, 0.372–10.759; *p* = .687), and heart failure (risk ratio, 0.929; 95% CI, 0.451–1.910; *p* = 1.000) (Table 2 *)*.

**Table 2:** Endpoints

Endpoint	Cilostazol (*N* = 154)	Aspirin (*N* = 154)	*p* value	RR (95% CI)
Primary endpoints				
Acute myocardial infarction	1 (0.6)	2 (1.3)	1.000	0.500 (0.046–5.457)
Nonfatal stroke	4 (2.6)	2 (1.3)	.687	2.000 (0.372–10.759)
Emerging arrhythmia	4 (2.6)	6 (3.9)	.727	0.667 (0.192–2.316)
Heart failure	13 (8.4)	14 (9.1)	1.000	0.929 (0.451–1.910)
All‐cause death	3 (1.9)	2 (1.3)	1.000	1.500 (0.254–8.852)
Secondary endpoints				
BARC 0	129 (83.8)	133 (86.4)	.636	0.970 (0.883–1.065)
BARC 1	24 (15.6)	18 (11.7)	.405	1.333 (0.755–2.355)
BARC 2	1 (0.6)	3 (1.9)	.625	0.333 (0.035–3.169)
BARC 3	0	0	‐	‐
BARC 4	0	0	‐	‐
BARC 5	0	0	‐	‐
Mucocutaneous adverse reaction^a^	9 (5.8)	9 (5.8)	1.000	1.000 (0.408–2.451)
Headache	4 (2.5)	6 (3.9)	.727	0.662 (0.191–2.301)
Gastrointestinal discomfort	7 (4.5)	9 (5.8)	.804	0.778 (0.297–2.036)

Abbreviations: BARC, Bleeding Academic Research Consortium; CI, confidence interval; RR, risk ratio.

^a^ Mucocutaneous adverse reaction refers to rash, urticaria, conjunctivitis, and angioneurotic edema.

### 3.4 Bleeding events

There were 129 (83.8%) and 133 (86.4%) BARC type 0 bleeding events (no bleeding events) in the cilostazol group and the aspirin group, respectively (risk ratio, 0.970; 95% CI, 0.883–1.065; *p* = .636). BARC type 1 bleeding events (inactive bleeding events that did not require medical attention, mainly spontaneous ecchymosis, nasal bleeding, spontaneous gingival bleeding, mild scleral bleeding) occurred in 24 (15.6%) patients in the cilostazol group and 18 (11.7%) patients in the aspirin group (risk ratio, 0.405; 95% CI, 0.755–2.355; *p* = .405). In terms of BARC type 2 bleeding events (significant active bleeding that requires medical intervention), only one patient (0.6%) from the cilostazol group and three patients (1.9%) from the aspirin group were recorded (risk ratio, 0.333; 95% CI, 0.035–3.169; *p* = .626). No significant BARC type 3 bleeding events (reduction in Hb, intracranial hemorrhage, intraocular hemorrhage, blood transfusion urgency), BARC type 4 bleeding events (CABG‐related bleeding events) nor BARC type 5 bleeding events (fatal bleeding) was recorded in either group. There was no significant difference in skin/mucosal adverse events (5.8 vs. 5.8%, *p* = 1.000) between the two groups, either were headache or gastrointestinal discomfort. Cilostazol combined with P2Y_12_ receptor inhibitors does not increase the risk of bleeding events and adverse reactions in people who are intolerant to aspirin (Table 2).

### 3.5 Survival analysis

The cumulative Kaplan–Meier was used to estimate the time to the first adjudicated occurrence of the composite MACCE points (acute myocardial infarction, nonfatal stroke, and all‐cause death) and major bleeding endpoints (BARC types 2–5). The risks of composite MACCE event (log‐rank *p* = .657) and major bleeding events (log‐rank *p* = .768) were similar in the cilostazol group and aspirin group (Figure S1).

Mean platelet volume (MPV), high‐sensitivity C‐reactive protein (hsCRP), estimated glomerular filtration rate (eGFR), triglycerides (TG), thrombin time (TT), prothrombin time (PT), D‐dimer, and calcium channel blockers were baseline variables statistically different between the two groups. To clarify whether these covariables had an impact on endpoints rates, Cox proportional hazards regression was conducted, using composite MACCE events and major bleeding events as dependent variables. The omnibus test showed a significance of 1.000 (>.05), demonstrating that MPV, hsCRP, eGFR, TG, PT, D‐dimer, and calcium channel blockers did not significantly impact composite MACCE events or major bleeding events.

## 4 DISCUSSION

Aspirin intolerance has become an increasing heightened risk with the emergence of rising coronary heart disease prevalence, especially in Southeast Asian populations.^24^, ^25^ In western countries, the prevalence of aspirin intolerance was estimated to range from 0.6% to 1.5%. However, a multicenter observational study in Japan involving 947 patients found that up to 30% of patients taking low‐dose aspirin clinically showed aspirin intolerance, while another cohort study in China revealed 9.9% of aspirin‐intolerant patients with CAD in one single center, mainly manifested as severe gastrointestinal reactions.^13^, ^25^, ^26^, ^27^ The concept of “Aspirin intolerance” has not been universally acknowledged before. It is often mislabeled as “aspirin hypersensitivity” or “aspirin resistance”.^28^, ^29^ Dai defined “aspirin intolerance” as “any conditions that prevent patients from long‐term use of low‐dose aspirin”,^13^ while earlier researchers conflated it with aspirin‐exacerbated respiratory disease.^28^ We summarized a clinical criterion of aspirin intolerance based on our previous cross‐sectional study and related published studies, which became the theoretical origin of inclusion criteria. Seven clinical criteria mainly covered gastrointestinal contraindications, hyperuricemia, and hypersensitivity (both respiratory and mucocutaneous). In this trial, it turned out that the prevalence of aspirin intolerance in percutaneous coronary stent implantation patients in four centers of Shanghai was 8.35% (172 out of 2059 patients).

Multiple meta‐analyses have shown that cilostazol‐based triple antiplatelet regimen can reduce the incidence of target vessel revascularization and target lesion revascularization after percutaneous coronary stent implantation.^30^, ^31^ However, the clinical outcomes and antiplatelet function of cilostazol plus P2Y_12_ receptor inhibitors have never been well detected before. Our trial was the first prospective multicenter noninferiority trial to evaluate the cilostazol‐based DAPT effects, which not only considered MACCE but also evaluated the mechanical suppression of PAC‐1, CD62p, and VASP‐P. We enrolled 154 aspirin‐intolerant patients and matched aspirin‐tolerant patients from successive 2059 percutaneous coronary stent implantation patients from four centers in Shanghai, China from January 2018 to January 2020. Our trial demonstrated that cilostazol‐based DAPT was not inferior to aspirin‐based DAPT on MACCE. In addition, there was no significant difference in all types of BARC bleeding events between the two groups. The antiplatelet effect reflected by the inhibition level of CD62p, PAC‐1, and PRI was similar in both groups. The substitute use of cilostazol plus P2Y_12_ receptor inhibitors in aspirin‐intolerant patients undergoing percutaneous coronary stent implantation reached noninferior efficacy and safety. In our trial, 172 out of successive 2059 patients undergoing percutaneous coronary stent implantation met the aspirin intolerance criterion. The primary manifestation was a history of peptic ulcer, atrophic gastritis, erosive gastritis, antral gastritis, and reflux esophagitis (33%), followed by high gastrointestinal bleeding events (27%). Remarkably, some patients were included in the cilostazol group with more than one intolerance factor, among which hyperuricemia was the most common comorbid factor (Table 3 and Figure S2). The mechanism of aspirin intolerance has not been well explained yet. Polymorphisms of enzymes like UDP‐glycolaldehyde transferase, cytochrome P450, and heterologous/medium‐chain fatty acid CoA ligase were thought to be the underlying mechanism of aspirin intolerance.^32^, ^33^

**Table 3:** Clinical manifestation of aspirin intolerance

Manifestation	*N*	%
1. History of peptic ulcer, atrophic gastritis, erosive gastritis, antral gastritis, and reflux esophagitis	57	33
2. High bleeding risk, active hemorrhage, and positive fecal occult blood during hospitalization (at least consecutive two times)	46	27
3. Gastric discomfort, acid regurgitation, and vomiting	31	18
4. Hyperuricemia and gout	14	8
5. History of subtotal gastrectomy or gastric polypectomy within 3 years	10	6
6. Hyperuricemia combined with peptic ulcer, bleeding, or gastritis	7	4
7. Aspirin‐exacerbated respiratory disease, including severe rhinitis, bronchospasm, and aspirin‐induced respiratory diseases	3	2
8. Hyperuricemia combined with gross hematuria	2	1
9. Hyperuricemia combined with asthma	2	1

Cilostazol is a specific and strong inhibitor of PDE3 in platelets and smooth muscle cells, where it diminishes intracellular calcium. In platelets, cilostazol inhibits both primary and secondary platelet aggregation induced by ADP, arachidonic acid, collagen, and adrenaline^9^; the addition of cilostazol to DAPT significantly decrease the level of P‐selectin expression, especially in patients with relatively high platelet activity.^34^, ^35^ Accordingly, we detected two downstream platelet activation signals, PAC‐1 and P‐selectin, to analyze the comprehensive inhibition effect of two combined antiplatelet drugs.^36^ The inhibitions of PAC‐1 and P‐selectin were similar in the two groups, which could help partly explain the final noninferior clinical efficacy of the substitute regimen. VASP‐P was recognized as an efficacy indicator of P2Y_12_ receptor inhibition.^19^, ^20^, ^37^ Besides, cilostazol was also found to have the ability to phosphorylate VASP through promoting PKA activation and further to inhibit platelet aggregation.^38^ It turned out that when cilostazol was combined with P2Y_12_ receptor inhibitors, similar phosphorylation of VASP was reached in two groups, indicating that there might be no cumulative effect of PDE III inhibitor and P2Y_12_ receptor inhibitors. It turned out that cilostazol combined with P2Y_12_ receptor inhibitors showed similar stronger phosphorylation of VASP.

Despite the encouraging findings, there were several limitations of this study. Though it was a multicenter trial, the population enrolled mainly came from southeast China, so transregional populations are needed to provide more convincing data. Our population with the number of 154 patients undergoing coronary stent implantation in each arm was relatively small. The missing harm made our study hard to draw a definitive conclusion or to apply to the overall population. Large‐scale randomized trials are still required in the future.

## 5 CONCLUSION

In patients with aspirin intolerance undergoing percutaneous coronary stent implantation, cilostazol combined with P2Y_12_ receptor inhibitors was not inferior to aspirin combined with P2Y_12_ receptor inhibitors on MACCE and BARC bleeding events. Similar inhibition of PAC‐1, CD62p, and VASP‐P was also found in the two groups. Cilostazol combined with P2Y_12_ inhibitors could be a reasonable substitute antiplatelet regimen for aspirin‐intolerant patients undergoing percutaneous coronary stent implantation, but again with limitations, which required a larger sample and longer follow‐up to confirm its efficacy.

## CONFLICT OF INTERESTS

The authors declare that there are no conflict of interests.

## AUTHOR CONTRIBUTIONS

*Conceived the original idea for the research*: Yikai Zhao, Xinping Luo, and Jian Li. *Patients' enrollment and data extraction*: Peng Zhou, Wen Gao, Haoxuan Zhong, and Yufei Chen. *Diagrams and data analysis*: Yikai Zhao, Wei Chen, and Kun Xie. *Revision of the tables and figures*: Maieryemu Waresi. *Drafting of the manuscript*: Yikai Zhao. *Manuscript revision*: Haiming Shi, Hui Gong, Guibin He, Zhaohui Qiu, Xinping Luo, Jian Li. All authors have read and approved the manuscript.

## Supporting information

Cumulative Kaplan‐Meier estimates of the time to the first adjudicated occurrence of the composite MACCE points and major bleeding endpoints. The risks of composite MACCE event (A, Log‐rank *P* = 0.657) and Major bleeding events (B, Log‐rank *P* = 0.768) did not differ significantly among the two groups.

Composition ratio of specific causes of the aspirin‐intolerant population.

Supporting information.

Supporting information.

## Data Availability

The data that support the findings of this study are available on request from the corresponding author. The data are not publicly available due to privacy or ethical restrictions.
