# Supplementary figures and images for: Cilostazol combined with P2Y12 receptor inhibitors: A substitute antiplatelet regimen for aspirin‐intolerant patients undergoing percutaneous coronary stent implantation
Source: Clin Cardiol. 2022 Feb 4;45(2):189–97. doi: 10.1002/clc.23787 (PMC8860475; doi:10.1002/clc.23787)

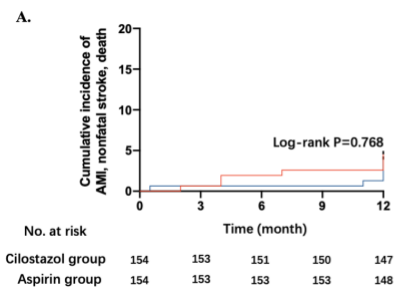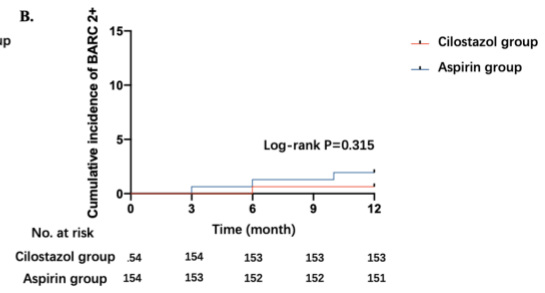

Supplement: Supplementary file 1 — Cumulative Kaplan‐Meier estimates of the time to the first adjudicated occurrence of the composite MACCE points and major bleeding endpoints. The risks of composite MACCE event (A, Log‐rank P = 0.657) and Major bleeding events (B, Log‐rank P = 0.768) did not differ significantly among the two groups. [file CLC-45-189-s001.pdf]

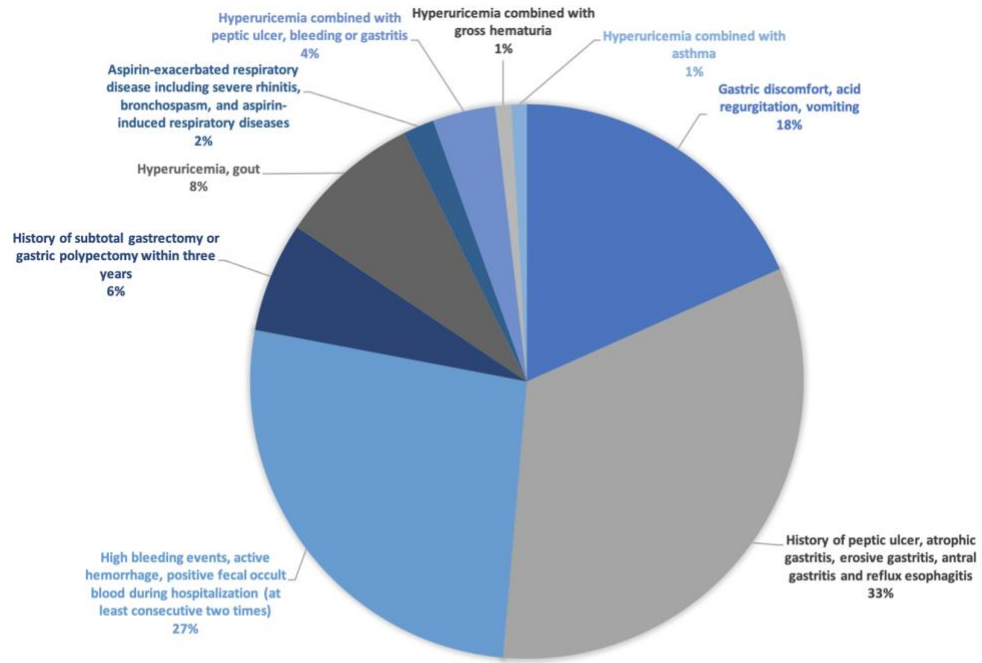

Supplement: Supplementary file 2 — Composition ratio of specific causes of the aspirin‐intolerant population. [file CLC-45-189-s003.pdf]
