# Supplementary material for: Cilostazol combined with P2Y12 receptor inhibitors: A substitute antiplatelet regimen for aspirin‐intolerant patients undergoing percutaneous coronary stent implantation
Source: Clin Cardiol. 2022 Feb 4;45(2):189–97. doi: 10.1002/clc.23787 (PMC8860475; doi:10.1002/clc.23787)
Supplement: Supplementary file 3 — Supporting information. [file CLC-45-189-s004.docx]

Supplementary table 1. Baseline characteristics before and after propensity score-matching.

| Variables | Before matching | |  | After matching | | *P* value |
| --- | --- | --- | --- | --- | --- | --- |
|  | Cilostazol  (N=160) | Aspirin  (N=1129) | *P* value | Cilostazol  (N=154) | Aspirin  (N=154) |  |
| Male | 121（75.6%） | 565（75.3%） | 0.938 | 115（74.7%） | 122（76.3%） | 0.419 |
| Age, years | 71（64～76） | 67（59～74） | **0.000** | 71（64～76） | 70（63～78） | 0.799 |
| Hypertension | 108（67.5%） | 489（65.2%） | 0.578 | 102（66.2%） | 95（61.7%） | 0.470 |
| Diabetes | 63（39.4%） | 243（32.4%） | 0.090 | 62（40.3%） | 56（36.4%） | 0.532 |
| CKD | 45（28.1%） | 90（12%） | **0.000** | 39（25.3%） | 36（23.4%） | 0.701 |
| Smoking | 62（38.8%） | 411（54.8%） | **0.000** | 62（40.3%） | 64（41.6%） | 0.888 |
| LDL-c | 2.01（1.53～2.64） | 2.19（1.72～2.82） | **0.016** | 2.02（1.55～2.66） | 2.06（1.57～2.62） | 0.895 |
| Clopidogrel | 123（76.9%） | 484（64.5%） | **0.003** | 117（76.0%） | 114（74.0%） | 0.761 |
| Ticagrelor | 37（23.1%） | 266（35.5%） | **0.029** | 37（24.0%） | 40（26.0%） | 0.766 |
