# Supplementary material for: Cilostazol combined with P2Y12 receptor inhibitors: A substitute antiplatelet regimen for aspirin‐intolerant patients undergoing percutaneous coronary stent implantation
Source: Clin Cardiol. 2022 Feb 4;45(2):189–97. doi: 10.1002/clc.23787 (PMC8860475; doi:10.1002/clc.23787)
Supplement: Supplementary file 4 — Supporting information. [file CLC-45-189-s002.docx]

Supplementary table 2. Flow cytometry test of PAC-1, CD 62p, and PRI% tested by VASP kit.

| Parameters | Placebo（N=10） | | Cilostazol（N=48） | | Aspirin（N=48） | | *P* |
| --- | --- | --- | --- | --- | --- | --- | --- |
|  | $\bar{x}$±s *(x*=arcsin$\sqrt{p}$) | $\bar{p}$  （%） | $\bar{x}$±s *(x*=arcsin$\sqrt{p}$) | $\bar{p}$（%） | $\bar{x}$±s *(x*=arcsin$\sqrt{p}$) | $\bar{p}$（%） |  |
| PAC-1% | 18.88±6.05 | 11.27 | 10.95±7.85 | 4.99 | 9.09±8.09 | 4.18 | 0.171 |
| CD62p% | 35.15±9.57 | 33.72 | 17.87±8.97 | 11.18 | 14.51±8.87 | 8.23 | 0.055 |
| PRI% | 61.72±14.90 | | 52.60±22.58 | | 49.51±23.76 | | 0.507 |
